# Smart water consumption measurement system for houses using IoT and cloud computing

**DOI:** 10.1007/s10661-020-08535-4

**Published:** 2020-08-28

**Authors:** Henry Fuentes, David Mauricio

**Affiliations:** Faculty of System Engineering and Informatic, Universidad Nacional Mayor de San Marcos, Calle Germán Amézaga N∘ 375, Lima, Perú

**Keywords:** Smart metering, Water consumption, IoT, Cloud computing

## Abstract

Presently, in several parts of the world, water consumption is not measured or visualized in real time, in addition, water leaks are not detected in time and with high precision, generating unnecessary waste of water. That is why this article presents the implementation of a smart water measurement consumption system under an architecture design, with high decoupling and integration of various technologies, which allows real-time visualizing the consumptions, in addition, a leak detection algorithm is proposed based on rules, historical context, and user location that manages to cover 10 possible water consumption scenarios between normal and anomalous consumption. The system allows data to be collected by a smart meter, which is preprocessed by a local server (Gateway) and sent to the Cloud from time to time to be analyzed by the leak detection algorithm and, simultaneously, be viewed on a web interface. The results show that the algorithm has 100% *Accuracy*, *Recall*, *Precision*, and *F1 score* to detect leaks, far better than other procedures, and a margin of error of 4.63% recorded by the amount of water consumed.

## Introduction

Water is the most important natural resource for humans, so the World Health Organization (WHO) recommends that a person should consume an average of 100 L per day to meet all their needs (United Nations 2014), but in the main capitals of South America it exceeds what is recommended, for example, La Paz consumes 120 L, followed by Bogotá with 168 L, then Santiago with 200 L, Quito with 220 L and, finally, Lima with 250 L, whose surplus is equivalent to more than 77 thousand Olympic water pools per year (SUNASS 2017). On the other hand, in the USA inside a house, the daily consumption of water is approximately 138 gallons (522 L), being the flush of the toilet where water is used the most (24%), followed by the faucets (20%), showers (20%), clothes washer (16%), leaks (13%), bathtubs (3%), dishwasher (2%), and others (3%) (DeOreo et al. 2016). Due to this excessive water consumption, water treatment supply companies are aiming to raise public awareness about the responsible use of water.

One of the functions of companies that supply water is to identify how and where waste is generated, which, in general, can be due to people’s neglect or leakage. Studies reveal that the amount of water wasted by leaks varies widely between different countries. In developed countries in Europe, it is approximately 15% (France: 27%, UK: 21%, Netherlands: 5%) (Growing Blue 2011) and in the USA 13% is estimated (DeOreo et al. 2016); on the other hand, some sub-developing countries have a high index that ranges from 20 to 70% (Sharma and Vairavamoorthy 2009). Other ways these companies use are the control and monthly billing of the consumption of this resource. In several countries, water consumption is not measured in real time, so the consumer must wait until the following month to receive the status of their consumption, which is generally obtained through “manual” measurements made at each house meter. This generates a daily lack of knowledge of water consumption and the inability to detect in time a non-visible leak that results in a waste of this resource and economic losses even for the supplier when this resource is subsidized. An alternative to this problem is the use of smart systems that can save to generate water from 2.8 to 10.0% (Liu and Mukheibir 2018); this avoids wrong measurements generated by human error, and it creates the trust in consumers in terms of receipts and water consumption payments (Joo et al. 2015), also it allows to report water leakage, which reduces the probability of reoccurrence by 50% (Schultz et al. 2018). Currently, there are several smart measurement systems that also help detect, predict, and alert in time any leakage or excess of water. Through the use of the rules, Water Balance, Minimum Night Flow (MNF), and statistical methods have come to predict 97% accuracy water leakage (Farah and Shahrour 2017), on the other hand, with machine learning methods reached 74% of accuracy (Patabendige et al. 2018). Also, through an algorithm that integrates rules MNF and Continuous Non-Zero Water Consumption (CNZ), achieved 98% accuracy (Luciani et al. 2019). However, none of these studies integrates at the same time the location, historical data, and rules such as MNF and CNZ, to detect possible water leaks, in addition, even the rate accuracy can be improved.

The integration of various aspects developed to detect water leaks are complemented in many situations, for example, the detection based on historical data is oriented by the consumption behavior and does not contemplate particular scenarios that can only be detected through rules, such as CNZ. Therefore, we are proposing a consumption measurement system and detection of water leakage integrating user location, historical data, and rules, as well, is based on an IoT architecture and cloud computing. The location allows to identify a possible leak if the user is not at home, the historical data of the user’s water consumption allows to find a pattern of daily consumption, the IoT architecture allows the capture and preprocessing of the consumption data of water obtained through smart meters, and finally, the analysis and visualization of this data is carried out in the Cloud.

The rest of the article is organized as follows. In “Literature review”, a full architecture review, wireless technology, security, and water leak detection algorithms. Subsequently, in “?? ??”, the system architecture with its main components is described together with the algorithm used to detect water leaks. Validation through numerical experiments and discussions are presented in “Results and discussions”. Finally, in “Conclusions”, conclusions are mentioned.

## Literature review

### Water leak detection

Farah and Shahrour (2017) conducted a study, where an intelligent measurement system is implemented to detect possible leaks in a university campus, and it is proposed to combine the Water Balance rules with Minimum Night Flow (MNF), which results in 97% accuracy, thus, is achieved to reduce the waste of water by leakage. In the research article by Schultz et al. (2018), a portal is implemented in a city of California where residents can monitor their water consumption; thus, for leak detection, it is proposed to establish based on an average (AVG) a limit (7.5 gph) of continuous water consumed during a 24-h period; likewise, its results show that users who used the system came to reduce by 50% the chances of having a leak again, but highlights that their methodology does not detect leaks less than the established limit. On the other hand, Farah and Shahrour (2018) demonstrated that using an automatic measurement system (AMR) for monitoring water been consumed, some water leakage can be detected quickly, for this they use a density probability function in order to identify regions of more or less probabilities of leakage based on data that was consumed before, during workdays, weekends, holidays, in this way, resulted they were able to detect 3 leaks in the Scientific Campus of the University of Lilledurante during 2015. In addition, Patabendige et al. (2018) observed that most commercial water consumption systems only provide basic statistics; however, they do not detect complex patterns of behavior of anomalous water consumption; therefore, they propose the use of the K-Nearest Neighbors (K-NN) algorithm to calculate the score anomaly for each day, and the results show that during one year they detected 31 days of leaks, where it was achieved and reached an accuracy of 74%. Finally, with the aim that people avoid wasting water due to leaks, an algorithm is proposed by Luciani et al. (2019) that detects leaks using the rules MNF and CNZ, which they reach a 98% accuracy.

### IoT architecture

The technological solutions for the measurement of water consumption are supported on an IoT architecture, and this refers to the design of the layers of the system that will allow communication between smart devices, together with analysis and decision-making based on the data collected of these devices (Lloret et al. 2016), for these reasons we review some of these architectures. In a study conducted by Lloret et al. (2016), an integrated IoT architecture is proposed that includes a review of the main features of smart meters and the existing communication protocols for smart measurement of electricity, water, and gas between different systems for a smart city. In a research article by Horsburgh et al. (2017), an open-source IoT architecture, which includes local processing and low-cost hardware, is proposed to measure and record water consumption in a university. Similarly, a low-cost IoT architecture is presented by Zafar et al. (2018), which in addition to its simplicity allows real-time monitoring of the temperature and humidity environment. Unlike the previous jobs consider one IoT device, in a study conducted by Stewart et al. (2018), an architecture is presented that includes water, electricity, and gas meters, which, through an information system, it shows the multi-dimensional behavior of the user. In relation to existing communication protocols, an architecture is presented by Alvisi et al. (2019) that includes an additional layer (Edge Gateway) that allows interaction between them, which the user can select from the variety of smart meters without having to feel tied to a supplier (vendor lock-in).

Some work on IoT architectures for intelligent water measurement has focused on quality. Dong et al. (2015) explore three major subsystems for smart water quality monitoring system, namely the data collection subsystem, data transmission subsystem, and data management subsystem. Saravanan et al. (2018) proposed a SCADA system that uses IoT to perform real-time monitoring, where temperature, color, flow, PH, and pressure are measured. Chen and Han (2018) desired to show the feasibility of collecting real-time data with high frequencies and instantly display them online within a smart city, for this they build a water monitoring system based on the platform “Bristol Is Open” and conclude that its architecture is easily scalable for a larger network of sensors.

### Wireless technology

Technological solutions based on IoT require wireless communication technologies, through which the devices can receive and send data effectively (Marais et al. 2016), so their review is necessary. In a research article by Joo et al. (2015), several intercom tests between IoT devices were performed, where it was determined that using UHF and the internet (TCP/IP) the signal is more stable compared with UHF, DCU, and Wibro, in addition, their results show that the average reception of the packages was 94.1%. On the other hand, to monitor and collect information (pH, temperature, etc.) of a river’s flow over a large area, in a study conducted by Chung and Yoo (2015), it is proposed to use a low-cost wireless sensor network (WSN) whose results show that data loss is below 1% and network traffic is reduced to 1/5. In addition, Marais et al. (2016) proposed to use ZigBee technology with mesh topology to build an extensive network of intercommunicated devices that allow maximum effectiveness when receiving and sending data and, based on their simulations, data loss resulted less than 0.14%. A water monitoring system is built by Chen and Han (2018) in a city based on the “Bristol Is Open” platform; likewise, Wi-Fi is used due to its long range (up to 100m) and a transmission packaged of up to 7Gbps.

## Smart water consumption measurement system

The smart measurement system is based on the development of an architecture for IoT that covers 5 important aspects. First, the capture of water consumption, which for security must have a data encryption mechanism (Zhu et al. 2018). Then, the local preprocessing of the consumption received. Third, the physical security of electronic devices. Then, the storage and visualization of the water consumption obtained. Finally, the analysis of consumption through the leak detection algorithm.

**Fig. 1:**
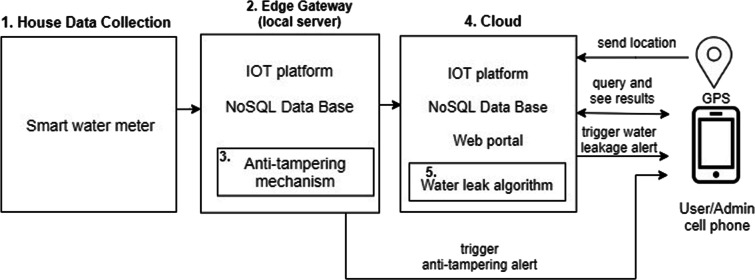
High-level diagram of the intelligent water system consumption measurement

Figure 1 shows the five main components of the system, which allow the collection, storage, analysis, and visualization of water consumption. In the “House Data Collection” component, each time period *t*_1_ (can be 1 min), the value of water consumption is obtained through a smart meter, which is sent to the “Edge Gateway” component for storage. Within this component there is an installed “Anti-Tampering” security mechanism that alerts the user and administrator in case of manipulation of the device. Then, each time period *t*_2_ (*t*_2_ > *t*_1_, it can be 1 h), the accumulated consumption is sent to the “Cloud” server so that this value is stored together with the user’s location, which is obtained through the cell phone’s GPS, and both are analyzed by the leak detection algorithm “Water leak Algorithm,” which alerts to the user and administrator if there is a possible water leak. Also, within the “Cloud” there is a web portal that allows the user to visualize, in real time, the history of their water consumption.

**Fig. 2:**
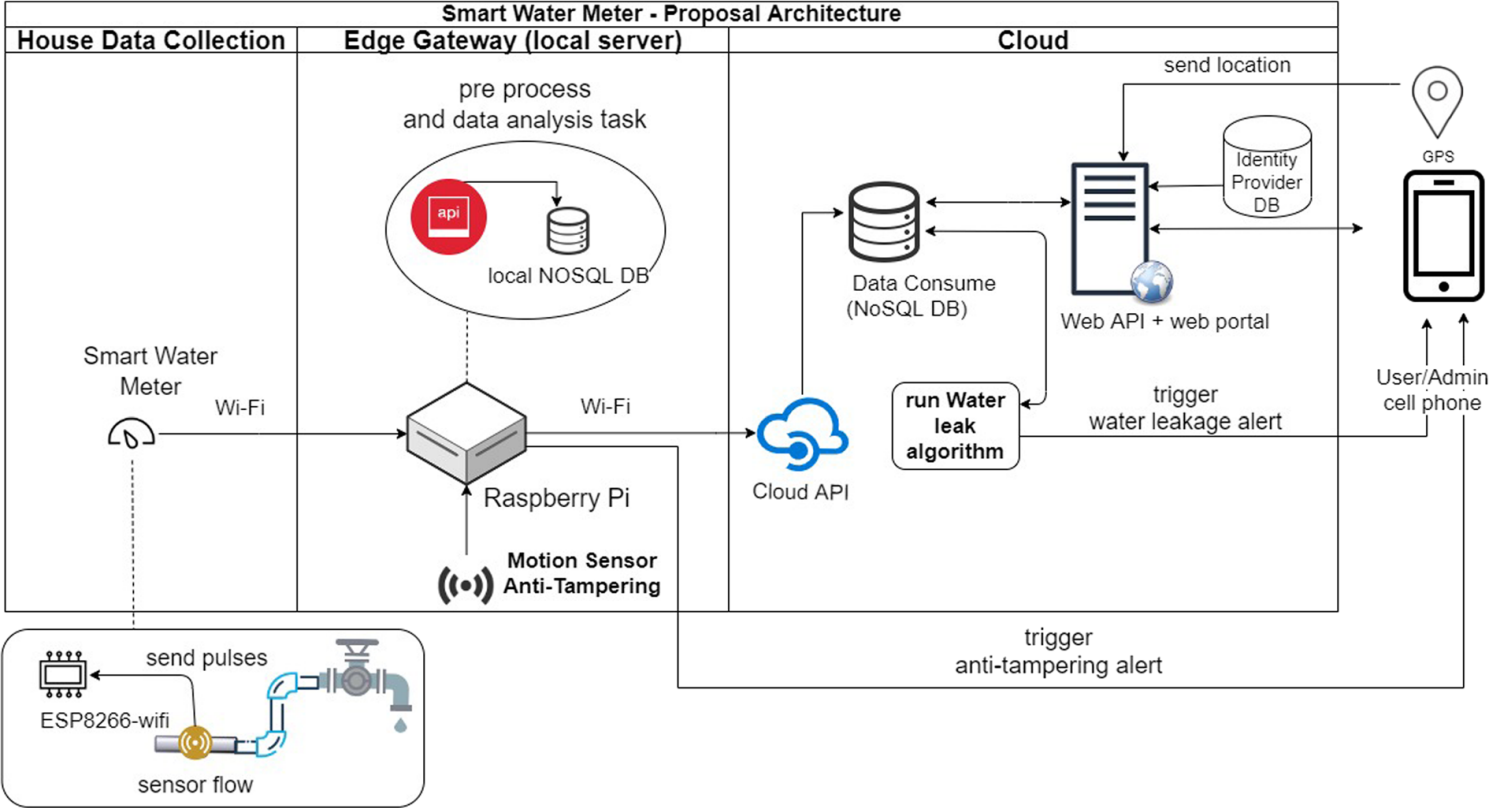
Physical view of the smart measurement of water consumption

**Fig. 3:**
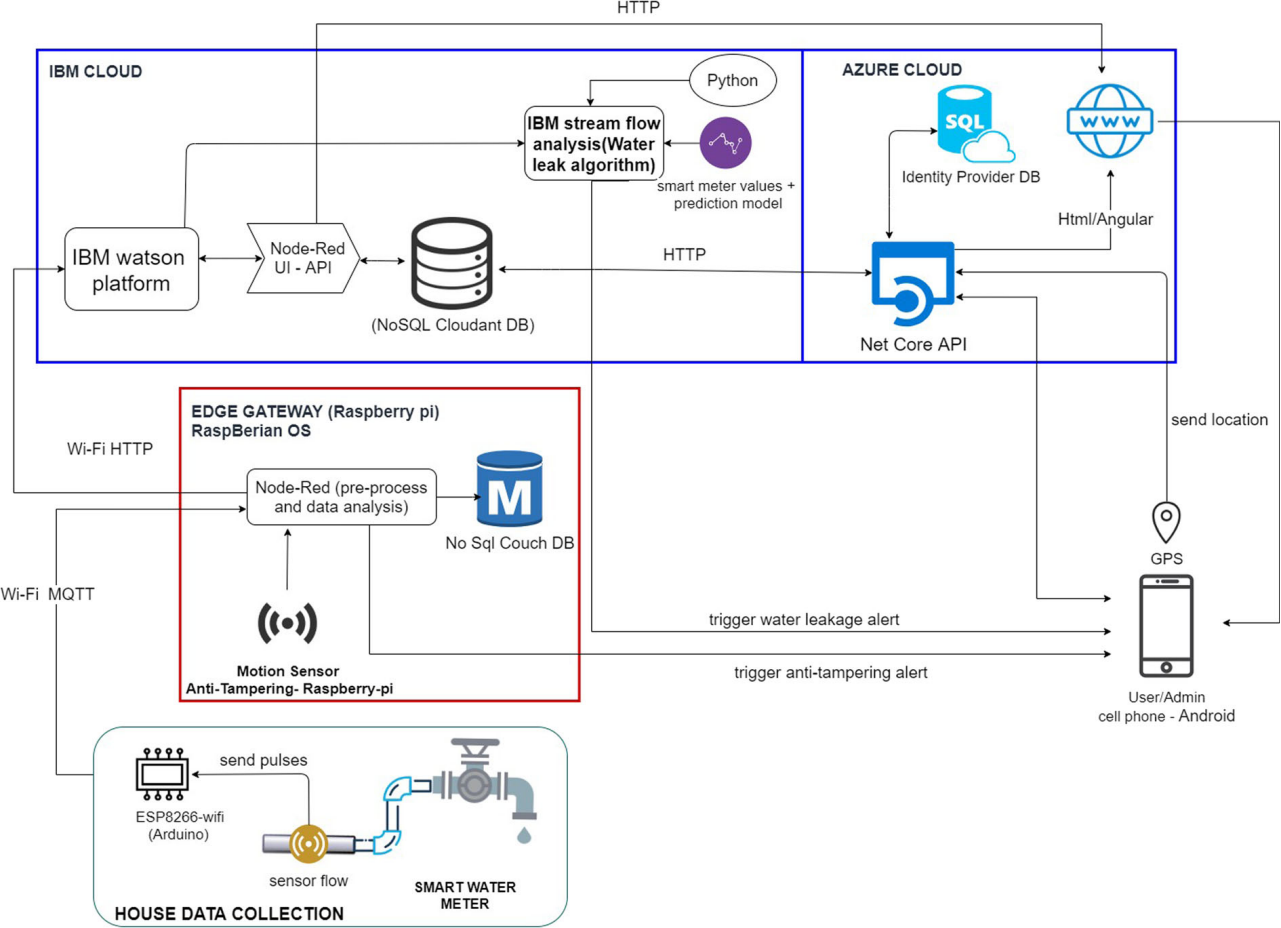
Technological view of the smart measurement of water consumption

On the other hand, in Figs. 2 and 3, the physical and technological view of the proposed architecture that connects the five components already mentioned can be appreciated. In the physical view, the physical devices used in each component together with the main services that are installed in them are shown in a high level. And in the technological view, it shows the name of the software, programming language, database, platform, and operating system used in each component.

### House data collection

**Fig. 4:**
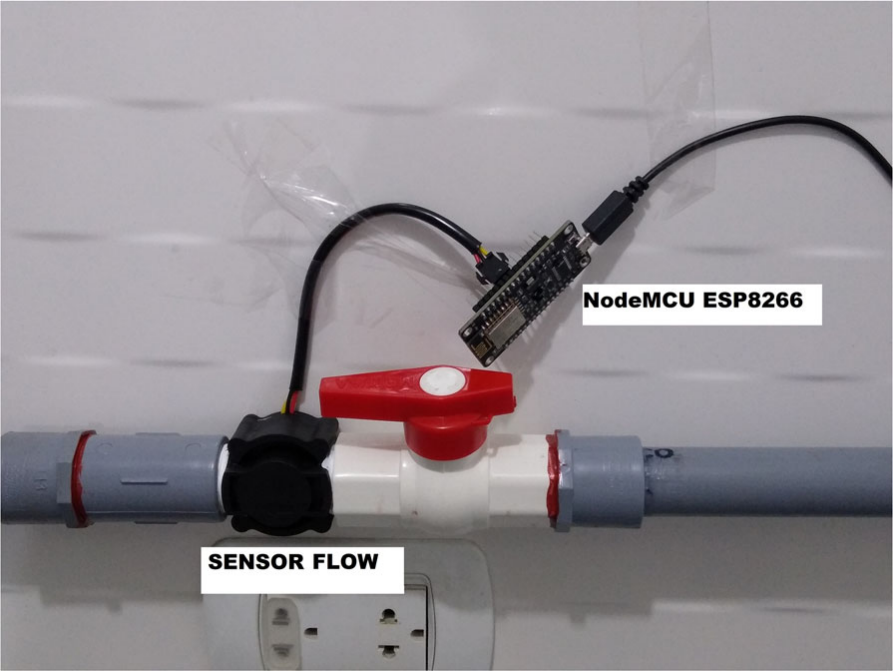
Smart water meter

Through this component, each time *t*_1_ captures water consumption, which is sent to the local server (Edge Gateway) digitally for storage and processing. The consumption is obtained through a sensor of water pulses (Seeed n.d.-a) , where approximately for every 367 pulses they are equivalent to the pass of 1 L of water. Then, the NodeMCU ESP8266 module (Handson Technology n.d.) is responsible for transforming these pulses to digital values with JSON format, which are sent to the Gateway via Wi-Fi and using a lightweight protocol called MQTT. Figure 4 shows the smart water meter used.

### Edge gateway

**Fig. 5:**
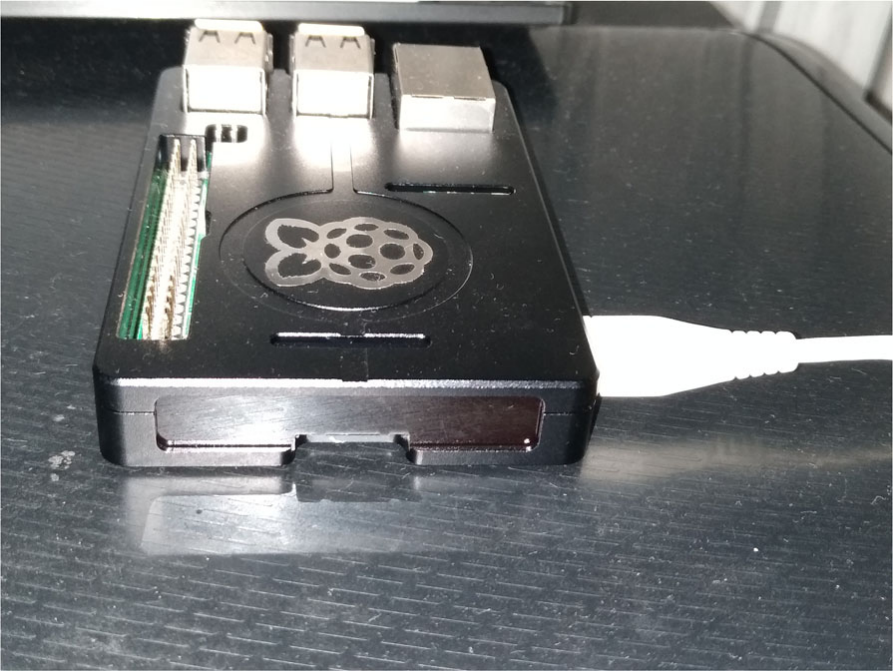
Raspberry Pi (Edge Gateway)

**Fig. 6:**
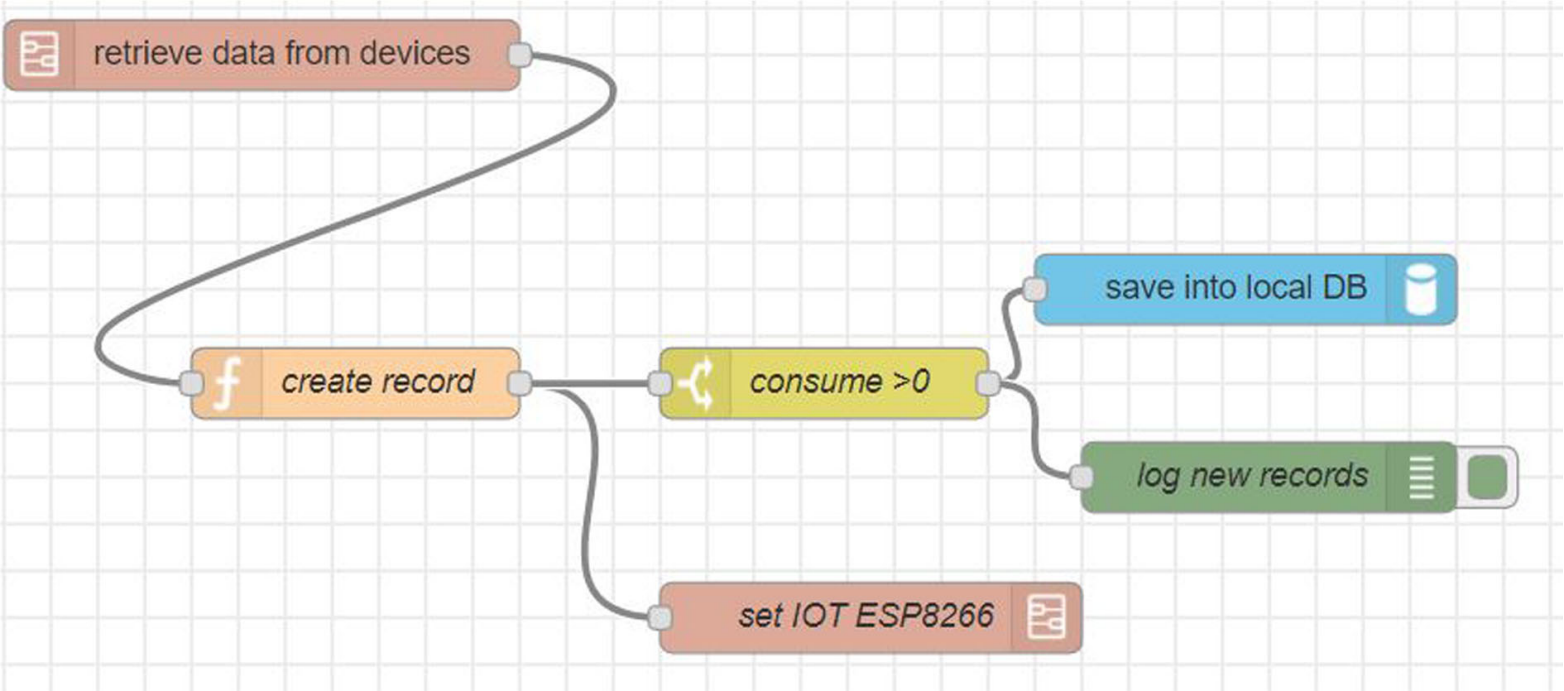
Node-Red—Flowchart of the data transformation and storage process

**Fig. 7:**
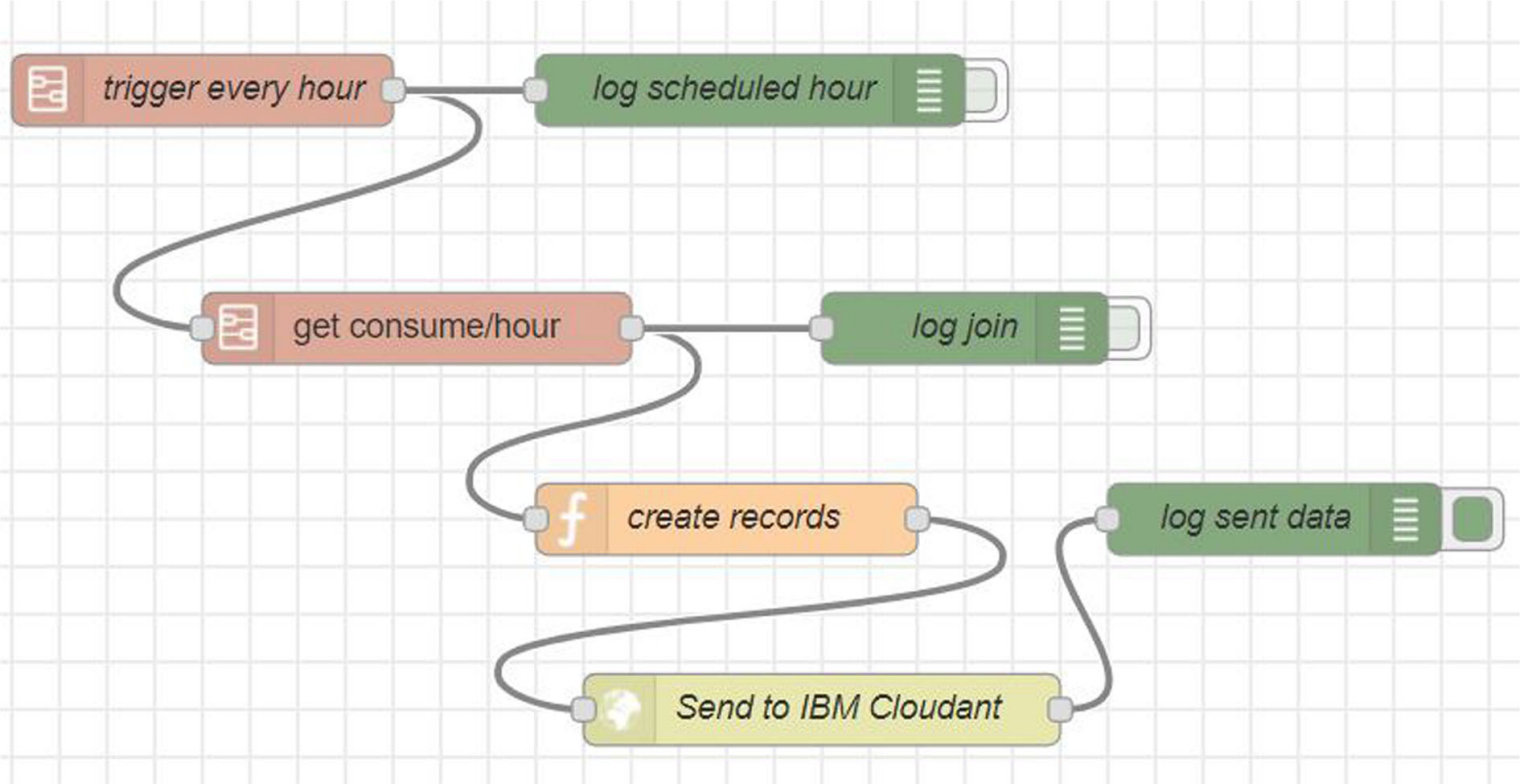
Flowchart of the data send process from the “Edge Gateway” component

This component receives the data obtained from the “House Data Collection” component, which are stored and processed to be subsequently sent in a single frame the accumulated in time *t*_2_ to the “Cloud” component. This local server is mounted on a small, low-cost computer with Wi-Fi connection called “Raspberry Pi” (Raspberry Pi n.d.), which is shown in Fig. 5. On the other hand, the processing is done using the “Node-Red” software, which, through a flowchart interface, adds logic that allows the transformation and storage of the data in a NoSQL database called CouchDB, as shown in Fig. 6, which contains a process that is executed every time *t*_2_, and which is responsible for obtaining the accumulated consumption within that period and sending it to the “Cloud” component for later storage and analysis (see Fig. 7, when *t*_2_= 1 h).

### Anti-tampering mechanism

This component seeks to ensure that, in case of any physical manipulation of the “Rasperry Pi” device, an alert is issued to the user and administrator in order to guarantee its proper functioning. This can be achieved through a vibration sensor such as SW-420 (Seeed n.d.-b), which also allows the sensitivity level to be calibrated. This component was not implemented in the prototype; however, Abreu et al. (2018) consider physical protection is a requirement of almost every IoT device that is physically accessible by anyone.

### Cloud

**Fig. 8:**
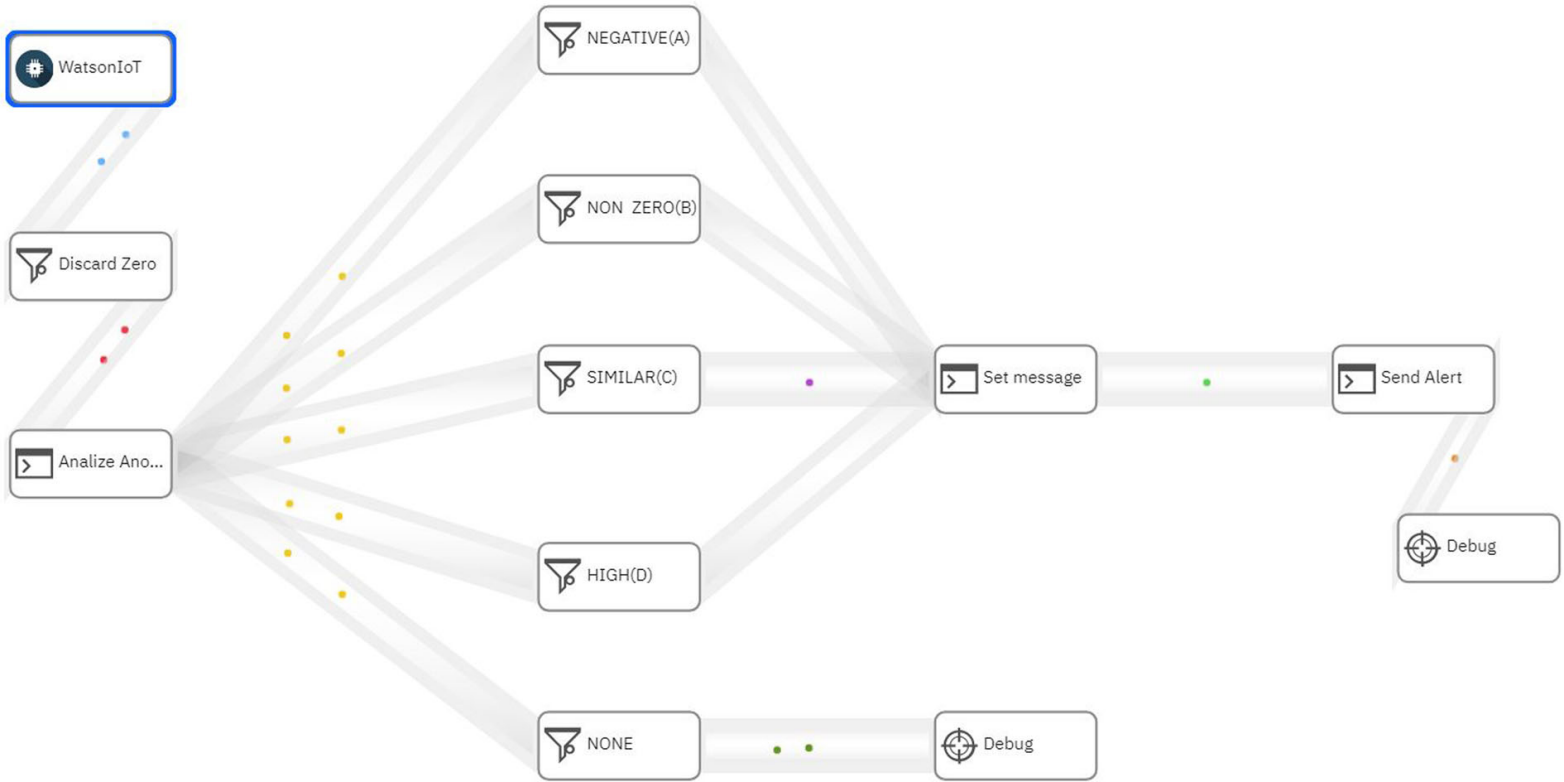
IBM stream flow analysis

**Fig. 9:**
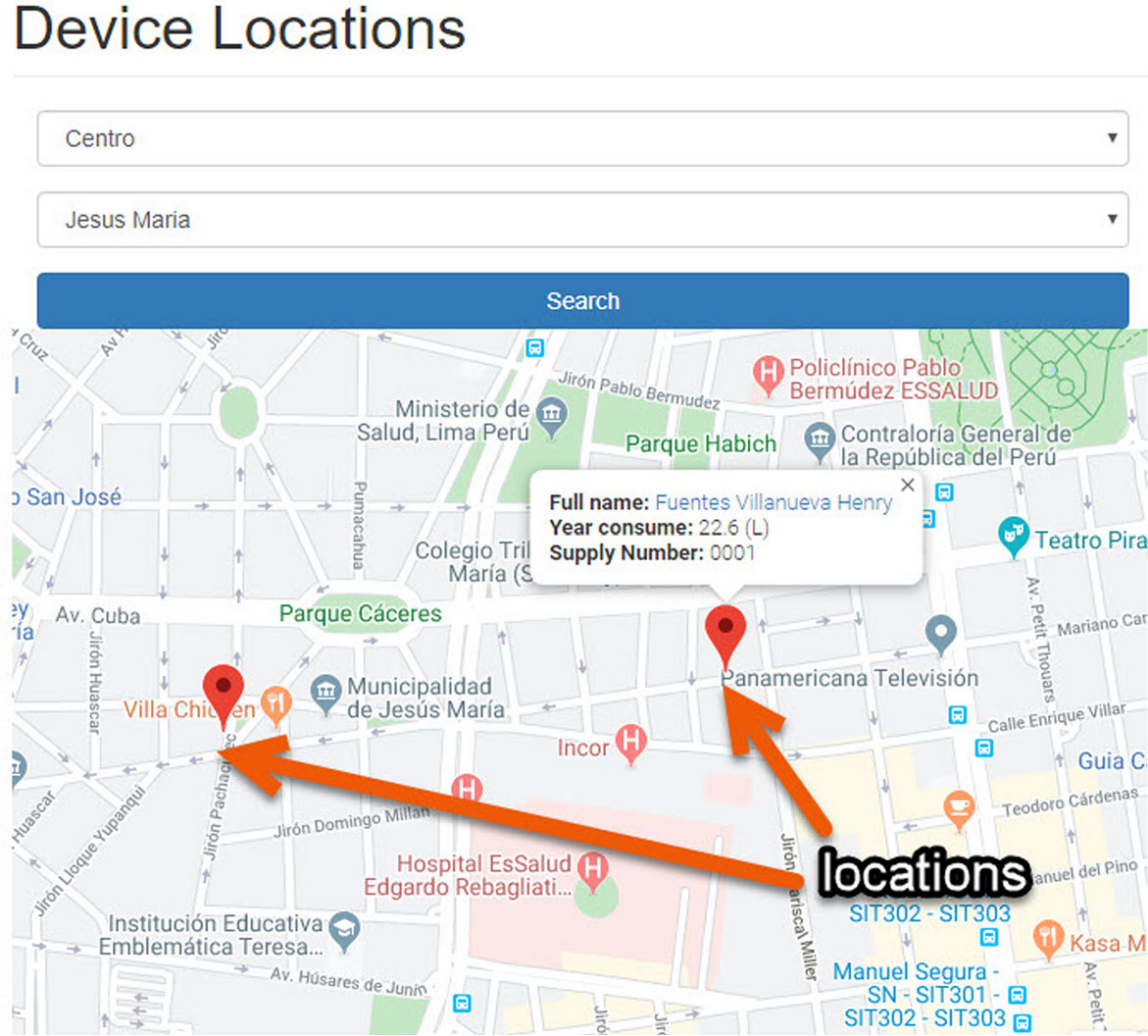
Device location view

**Fig. 10:**
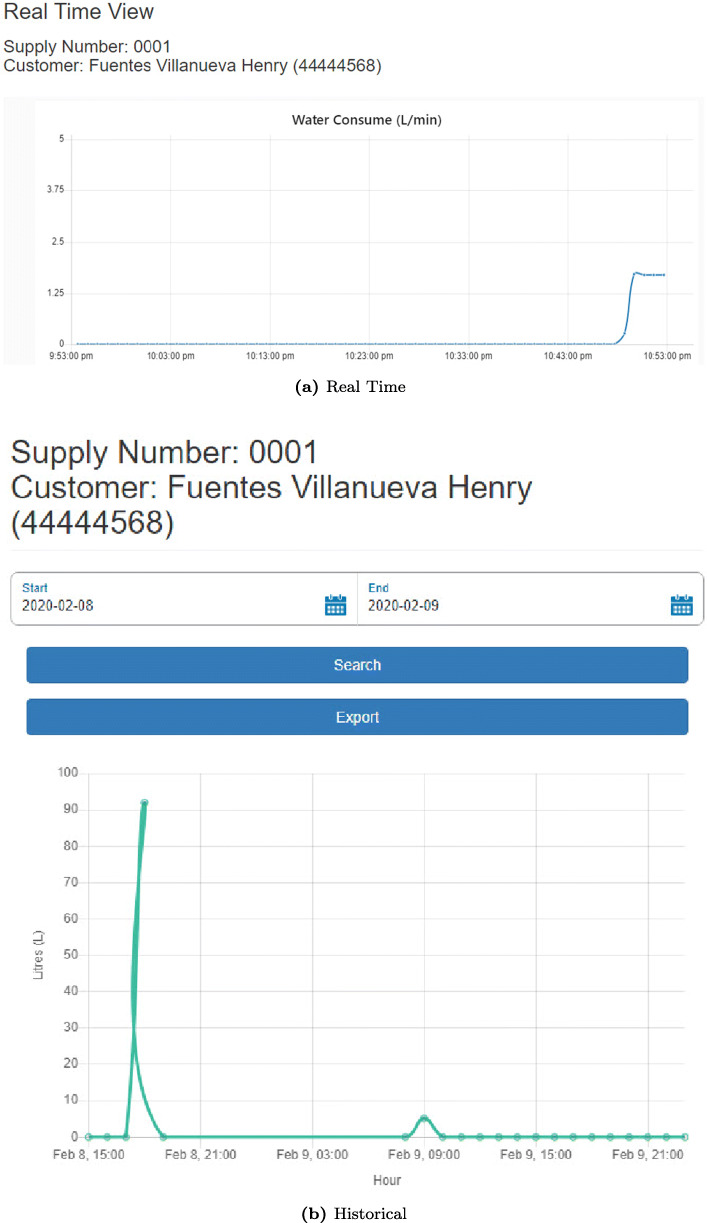
Consumption views

This component receives the data obtained from the “Edge Gateway” component and the user’s location through the cell phone’s GPS, which is sent every time *t*_3_ (*t*_1_ < *t*_3_ < *t*_2_), so that they are jointly stored, analyzed, and displayed in a Cloud platform. Storage is done in a NoSQL database called “Cloudant” from IBM Cloud (IBM n.d.-b). Then, the analysis is performed on the IBM Stream Flow Analysis platform (IBM n.d.-a), which allows analyzing and acting in real time on massive amounts of data (structured or unstructured) that may come from different sources and that are constantly sent, such as shown in Fig. 8, and under an algorithm made in Python, it instantly evaluates each record that arrives to see if there is a possible leak. In addition, in this component “Cloud” there is a web portal, which is deployed in Azure Cloud (Microsoft n.d.), so that users can see, in real time, the location of their smart meters, which are obtained by GPS from the users’ cell phone at the time of installation, and their water consumption (in real and historical time), as shown in Figs. 9 and 10, respectively, through an interface made in Angular, which obtains the data from an API programmed in .NetCore that connects to the “Cloudant” database through the http protocol.

### Water leak detection algorithm

**Fig. 11:**
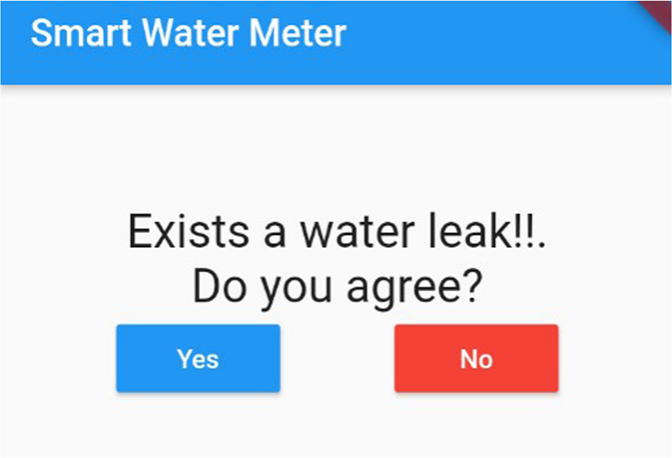
Alert and confirmation message

**Fig. 12:**
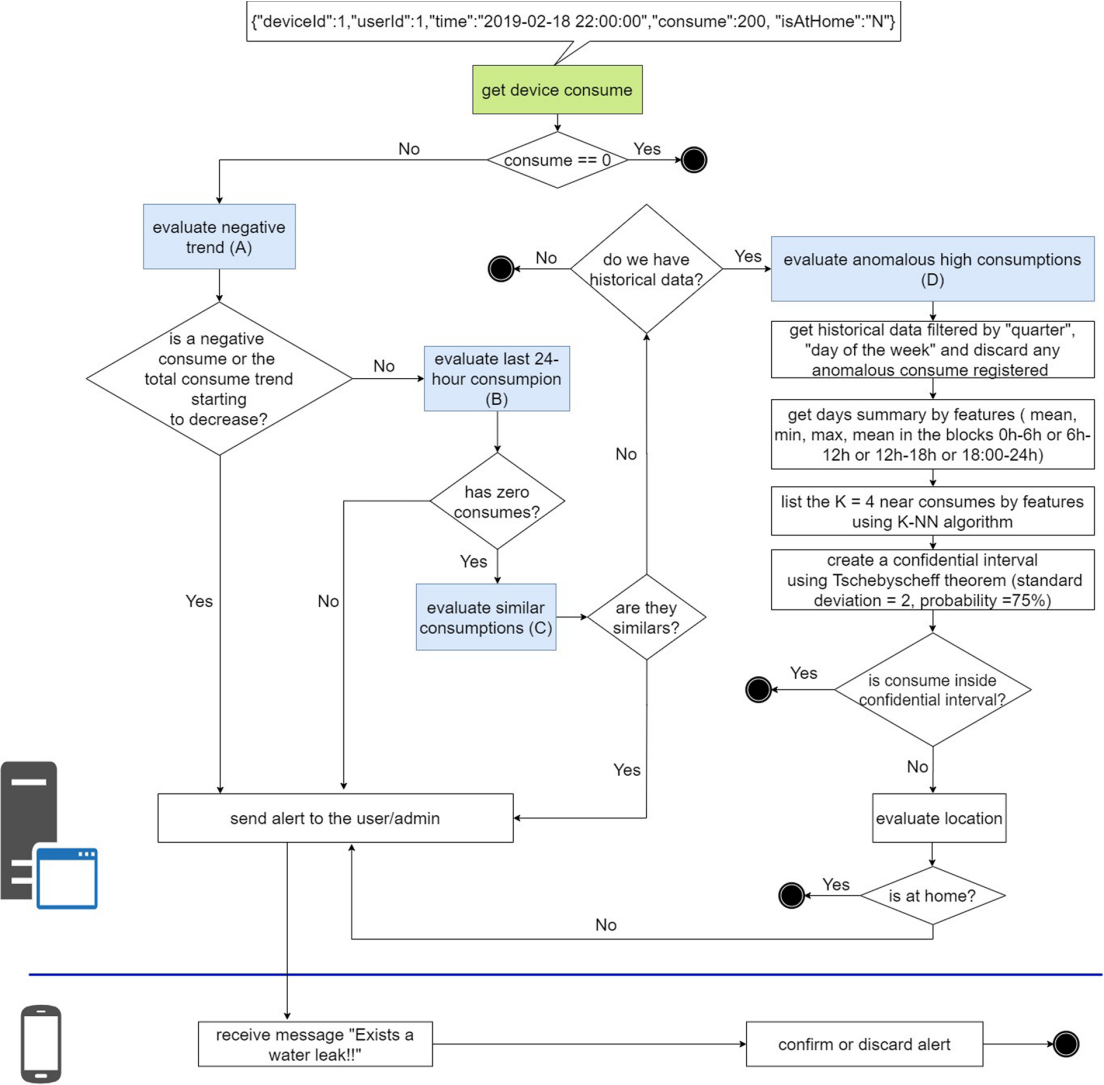
Flowchart of the leak detection algorithm

The algorithm shown in Fig. 12 detects the possible existence of a water leak considering four scenarios, for this it takes the input parameters: device ID, user ID, time *t*_2_, consumption in *t*_2_, and the location, the latest was used in the article conducted by Pan et al. (2015) to reduce consumption in smart homes. Each iteration of the algorithm is executed at the end of *t*_2_, and it checks whether the water consumption matches any of the four leak scenarios: “negative trend” (A), “24-hour consumption” (B), “similar consumptions” (C), and anomalous high consumptions” (D). This verification of scenarios is carried out sequentially A, B, C, D, and if at least one of them is verified, a leak detection alert is sent to the user and the administrator, who can confirm or reject the alert through the mobile application (see Fig. 11), and thus improves the precision of the algorithm in a subsequent iteration. Each scenario is explained below:

- **Scenario A** verifies if the consumption received has a negative value or the total consumption accumulated in the last 24 h has a negative trend; this could be due to failures in the smart meters when capturing consumption (Alvisi et al. 2019).
- **Scenario B** verifies if there is a continuous flow of water consumption in the last 24 h, since there was no consumption at any zero time, which is highly unlikely for normal consumption; this rule is known as CNZ.
- **Scenario C** verifies if the consumption received coincides with the last two registered consumptions, since it is highly unlikely that consecutively there will be very similar consumptions; we call this rule C3S (three similar consumptions in a row).
- **Scenario D** verifies if there is a high consumption outside its historical behavior. To do this, first all historical consumption that resembles this is obtained, both in the quarter, day of the week, and after, all those that have been marked as anomalous. Afterwards, these consumptions are indexed by days, and each day follows four characteristics that are obtained: average consumption, minimum consumption, maximum consumption, and the average hour range to which the consumption being evaluated belongs; this range can be between 0 h to 6 h, 6 h to 12 h, 12 h to 18h and from 18 h to 24 h. Next, for each of the four characteristics, the K-NN algorithm is applied in order to obtain a list of the consumptions that are closest (*K* = 4) to the input consumption. Next, the Tchebysheff theorem (Barnes 1994) is used to construct a confidence interval, which guarantees that at least 75% of the list of consumption obtained previously is within 2 standard deviations of the mean, if the value of consumption received is outside this range, it is considered a “possible leak” of water. Finally, if there is “possible leakage” and the person is not at home, consumption is considered high outside of their historical behavior. This entire sequence of steps has been denoted as the CHA (historical anomalous consumption) rule.

## Results and discussions

The proposed solution was installed in the department of the city of Lima, and was evaluated in two different aspects. First, the error rate of the water consumption record reported by the system was evaluated. Secondly, the leak detection algorithm accuracy was measured where a data set was used and the consumptions were simulated to see if the application detected or not a possible leak (Fig. 12).

**Fig. 13:**
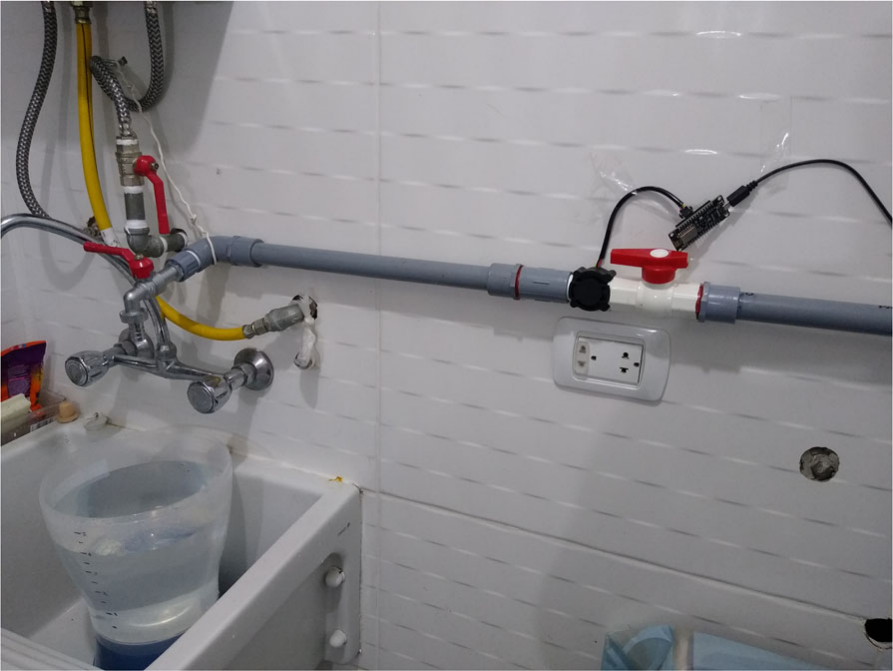
Smart meter model

To evaluate the margin of error of the smart meter on water consumption in liters, a model had to be assembled and the water flow measurement algorithm was gradually calibrated. Figure 13 shows the design of the model, where the water flow is measured by a flow sensor that records the pulsations generated by the passage of water. Then, through a bucket with marks (0.5 L, 1 L, 1.5 L, 2 L, 2.5 L, 3 L, 3.5 L, 4 L, 4.5 L, and 5 L), the liters registered by the system were corroborated against the actual liters that have passed through the pipe. It is worth mentioning that the calibration started with the factor recommended by the sensor documentation (Chung and Yoo 2015), which details that 330 pulses/min equivalent to 1 L, but, because the margin of error was very high with that factor, it continued calibrating until reaching 372 pulses/min. The margin of error is calculated with the following metric:

1 $$ Error =| (real value-value) / real value*100)| $$

Table 1 shows the ten tests carried out with the last mentioned factor and reached a percentage of 4.63% error margin, for 10 random values from 0.5 to 5 L.

**Table 1:** Comparison of actual water consumption vs. consumption recorded by the system

	Pulses	Real value (liters)	Value (liters)	Error
1	367	1.00	0.99	1.00%
2	546	1.50	1.50	0.00%
3	794	2.00	2.14	7.00%
4	1153	3.00	3.10	3.33%
5	1197	3.00	3.22	7.33%
7	1504	4.00	4.04	1.00%
8	1606	4.00	4.32	8.00%
9	1812	4.50	4.87	8.22%
10	1967	5.00	5.29	5.80%
Avg.				*4.63%*

On the other hand, to measure the precision of the leak detection algorithm, 10 different scenarios were simulated, this being compared with other existing algorithms. In first place, the test data was obtained (DAIAD 2019) that serves to obtain a history of consumption, and it consists of 674,020 records of 92 consumers, in 1 year of consumption, with hourly consumption records, and in many cases less than 24 records per day; because the data is desired to be the most recent, the measurement dates were updated to the years 2018–2019 and only 9 consumers were randomly selected, generating a dataset of 69,194 records. Subsequently, the following scenarios were defined:

- **Normal Consume Week (NCW):** These are the hourly consumptions between Monday and Friday where there is a normal consumption of water without the presence of a leak.
- **Normal Consume Weekend (NCWD):** These are the hourly consumptions between Saturday and Sunday where there is normal water consumption without the presence of a leak.
- **Normal Consume Night Work (NCNW):** These are the hourly consumption on the days where a person usually does work at dawn and his water consumption is considered normal.
- **Normal Consume First Day (NCFD):** Refers to hourly consumption on the first day of system use, where there should be normal consumption.
- **Normal High Consume Is at Home (NHCIAH):** Consumption per hour on days where there was a high increase in water consumption, but the user is at home and is not considered an anomaly or water leak.
- **Anomalous High Consume Week (AHCW):** These are the hourly consumptions between Monday and Friday where there is the presence of leakage due to high anomalous consumption.
- **Anomalous High Consume Weekend (AHCWD):** These are the hourly consumptions between Saturday and Sunday where there is the presence of leakage due to high anomalous consumption.
- **Anomalous Consume Non-Zero (ACNZ):** These are the hourly consumptions in which during the last 24 h in a row water consumption has not stopped registering and there is not at least 1 h where consumption is zero.
- **Anomalous Consume Similar (ACS):** These are the hourly consumptions where there are three consecutive consumptions with very similar values (+ -1 L), which is considered anomalous.
- **Anomalous Consume Negative (ACN):** These are the hourly consumptions in which during the last 24 h there has been a negative trend in the accumulated consumption of water or a negative consumption has been registered.

The algorithms applied are the Minimum Night Flow (MNF), Continuous Flow (CF), and Average per Hour (AVG). The MNF assumes that any existing water consumption between 2:00 a.m. and 4:00 a.m. they are indications of a possible leak. Then, the CF says that if there is no zero consumption within a 24-h range, it is considered a possible leak. Finally, the AVG is an average of consumption per hour made and if that average is passed it is an indication of leakage. To simulate anomalous consumption, consumption had to be updated at certain times after 2019, but the data for 2018 were not altered in order to have a historical pattern of behavior that would help us detect any anomalous behavior in 2019. The alterations were made to generate records for the anomalous scenarios and the NHCIAH scenario, for example, for the ACNZ scenario, a random value was added to consumption that had zero, and for NHCIAH it was established that the user was within their home having a high consumption. The data set and test scenarios are available at https://github.com/henrygustavo/data_set, with the test distribution by scenario of NCFD with 48, NCNW with 32, NCW with 120, NCWD with 46, NHCIAH with 29, ACN with 68, ACNZ with 96, ACS with 32, AHCW with 85, and AHCWD with 48, making a total of 275 tests for scenarios of normal consumption and 329 for anomalous consumption. In addition, each test has a field called “isAnomalous” with a value of 1 or 0 that indicates whether or not to issue a leak alert for a specific consumption.

The measurement of the algorithm is carried out through the confusion matrix that allows measuring the performance of the algorithms against the reference or expected consumption (Benchmark), which is appreciated in Table 2, where its main values are:

- **True Positive (TP):** Water leak identified by the algorithm.
- **False Positive (FP):** Non-existent water leak, incorrectly identified by the algorithm (false alarm).
- **False Negative (FN):** Water leak not identified by the algorithm.
- **True Negative (TN):** Real absence of water leakage (most cases).

**Table 2:** Confusion matrix

		Benchmark	
		Presence of water loss	Absence of water loss
Algorithm	Presence of water loss	TP	FP
	Absence of water loss	FN	TN

The following metrics were calculated based on the results provided by the confusion matrix:

2 $$ Accuracy = \frac{TP+TN}{TP+TN+FP+FN} $$

3 $$ Recall = \frac{TP}{TP+FN} $$

4 $$ Precision = \frac{TP}{TP+FP} $$

5 $$ F1 score = 2 \times\frac{Precision \times Recall}{Precision + Recall} $$

*Accuracy* indicates the percentage of leak and non-leak scenarios correctly identified by the algorithm. *Recall* quantifies the algorithm’s ability to identify alarms, measured by the ratio, correctly identified alarms to the numerical total of true alarms. *Precision* measures the algorithm’s ability to avoid false alarms, based on the ratio between the number of identified true alarms and the total number of alarms identified by it. Finally, the *F1 score* allows evaluating the algorithm’s ability, in a single metric, to distinguish between hours with and without water loss and is calculated as the *Recall* and *Precision* harmonic mean.

Tables 3 and 4 show the results obtained from the tests carried out in the different scenarios of normal and anomalous consumption, respectively. The “# Tests” column is the amount of consumption per hour that has been tested by the different algorithms on a given day. Then, the “Leakage / day” column is the number of leaks to be detected on a given day. Subsequently, the columns “MNF”, “CF”, “AVG”, and “Proposed Algorithm” show the number of leaks that have been detected during the day.

**Table 3:** Scenarios of normal water consumption

Scenario	UserId	Date	# Tests	Leakage/day	MNF	CF	AVG	Proposed algorithm
NCFD	2	2018-03-01	24	0	0	0	0	0
NCFD	3	2018-03-01	24	0	2	0	0	0
NCNW	1	2019-02-25	16	0	3	0	1	0
NCNW	1	2019-02-27	16	0	3	0	1	0
NCW	2	2019-01-07	24	0	0	0	5	0
NCW	2	2019-01-22	24	0	1	0	4	0
NCW	2	2019-01-23	24	0	0	0	5	0
NCW	3	2019-01-24	24	0	2	0	3	0
NCW	3	2019-02-08	24	0	4	0	1	0
NCWD	4	2019-02-02	24	0	1	0	1	0
NCWD	4	2019-02-24	22	0	1	0	2	0
NHCIAH	1	2019-02-01	9	0	0	0	6	0
NHCIAH	4	2019-01-07	20	0	0	0	1	0
Total			*275*	*0*	*17*	*0*	*30*	*0*

**Table 4:** Scenarios of anomalous water consumption

Scenario	UserId	Date	# Tests	Leakage/day	MNF	CF	AVG	Proposed algorithm
ACN	7	2019-02-28	22	1	0	0	5	1
ACN	8	2019-02-28	23	1	1	0	1	1
ACN	9	2019-02-28	23	2	1	0	2	2
ACNZ	3	2019-01-02	24	1	5	1	4	1
ACNZ	3	2019-01-18	24	1	5	1	5	1
ACNZ	7	2019-01-17	24	1	5	1	6	1
ACNZ	9	2019-01-15	24	1	5	1	6	1
ACS	1	2019-01-04	4	1	3	0	0	1
ACS	1	2019-02-02	20	1	0	0	5	1
ACS	8	2019-01-03	8	1	0	0	3	1
AHCW	1	2019-02-21	8	1	0	0	1	1
AHCW	5	2019-02-27	16	1	3	0	7	1
AHCW	5	2019-02-28	22	2	3	0	12	2
AHCW	6	2019-01-15	20	1	0	0	3	1
AHCW	6	2019-02-18	19	1	0	0	6	1
AHCWD	8	2019-02-23	24	1	0	0	1	1
AHCWD	8	2019-02-24	24	1	0	0	1	1
Total			*329*	*19*	*31*	*4*	*68*	*19*

Table 5 shows the result of the confusion matrix, where it can be seen that the proposed algorithm has an *Accuracy*, *Recall*, *Precision*, and *F1 score* of 100% that are superior to the other algorithms.

**Table 5:** Results of the confusion matrix by the algorithm

Metric	Metric name	MNF	CF	AVG	Proposed algorithm
Population	Population	604	604	604	604
P	Condition positive	19	19	19	19
N	Condition negative	585	585	585	585
TP	True Positive	1	4	10	19
TN	True Negative	538	585	497	585
FP	False Positive	47	0	88	0
FN	False Negative	18	15	9	0
ACC	Accuracy	0.89	0.98	0.84	1.00
TPR	Recall	0.05	0.21	0.53	1.00
PPV	Precision	0.02	1.00	0.10	1.00
F1_score	F1 score	0.03	0.98	0.17	1.00

## Conclusions

This work has proposed an architecture for a water consumption measurement system that covers five important aspects, which are the measurement of water consumption, local record consumption process, physical security of the electronic device, storage and visualization of the consumption obtained, and leak detection. The proposed architecture is functional, integral because it covers various aspects of measurement and leak detection, and is maintainable due to its high level of decoupling. In addition, a system based on the proposed architecture has been implemented which shows that the architecture allows the integration of various technologies and programming languages, the communication between different Cloud services, the use of low-cost hardware, the use of free software, and also covering various aspects of the problem of water consumption measurement and leak detection.

For the leak detection, an algorithm was introduced that considers the location of the user, the historical data, and the rules MNF and CNZ of the literature and C3S and CHA are the proposed rules that manage to cover 10 possible scenarios of water consumption between normal and anomalous consumption. Numerical tests on 10 records of water consumption show that the system has a margin of error of 4.63% with low-cost equipment. Also, the leak detection algorithm for 604 test cases between normal and anomalous detects all leak situations, and presents an *Accuracy*, *Recall*, *Precision*, and *F1 score* of 100%, surpassing the rest of the leak detection algorithms. Finally, future work will be focused on two important areas such as device security and the architecture scaling for microservices, which we consider feasible given the high decoupling of the components.
